# Radial extracorporeal shockwave therapy (rESWT) for coccydynia: a prospective study of 14 patients

**DOI:** 10.1097/MS9.0000000000001133

**Published:** 2023-08-04

**Authors:** Kabir Singh Lota, Nikos Malliaropoulos, Georgios Bikos, Heinz Lohrer

**Affiliations:** a Barts and The London School of Medicine and Dentistry; b Centre for Sports and Exercise Medicine, Queen Mary, University of London; c Sports Clinic, Rheumatology Department, Barts Health NHS Trust, London, UK; d Sports and Exercise Medicine Clinic; e Euromedica – Arogi Rehabilitation Center, Thessaloniki, Greece; f European Sportscare Network (ESN), Zentrum für Sportorthopädie, Wiesbaden-Nordenstadt; g Department of Sport and Sport Science, Albert-Ludwigs-Universität Freiburg i. Brsg., Freiburg, Germany

**Keywords:** adapted, coccydynia, conservative, ESWT, shockwave

## Abstract

**Background:**

Coccydynia is defined as pain in the coccyx. We investigated the effect of radial extracorporeal shockwave therapy (rESWT) in the management of coccydynia.

**Methods:**

In this prospective study, patients (≥18 years) diagnosed with coccydynia at a sports clinic located in Thessaloniki, Greece, were eligible for rESWT treatment when they reported a visual analogue scale (VAS) pain level ≥6. Treatment sessions were once weekly and ended when VAS pain levels decreased to ≤3. Recurrence rates were documented at 3-month and 12-month follow-ups.

**Results:**

Fourteen patients were treated using rESWT. The mean age and symptom duration of our cohort was 33.6±7.9 (range: 20–45) years and 9.4±8.5 (range: 3–36) months, respectively. The mean number of treatment sessions per patient was 6.4±1.6 (range: 4–8). The mean device pressure, frequency, and number of pulses was 1.2±0.1 (range: 1–1.4) bar, 5.0±0.1 (range: 5–6) Hz, and 2082±74.8 (range: 2000–2300) pulses, respectively. Treatment alleviated pain in all patients, and no recurrence of symptoms was reported during follow-up. There was a positive correlation between symptom duration and the number of treatment sessions (*r*=0.701, *P*=0.005). Pairwise comparison highlighted significant reductions in VAS pain levels between each stage of treatment (*P*<0.001).

**Conclusion:**

Our study affirms the safety and efficacy of rESWT in managing coccydynia.

## Introduction

Highlights

- Radial extracorporeal shockwave therapy (rESWT) offers an alternative and non-invasive means of treatment for coccydynia.
- Treatment can be adapted by altering device parameters in line with patients’ pain tolerance and treating patients over a variable number of sessions.
- Pain-adapted intervention protocols may optimise pain management and lower the risk of symptom recurrence.
- Randomised controlled trials comparing rESWT efficacy with other surgical and non-surgical modalities are needed.

Coccydynia, or coccygodynia, refers to pain in the coccyx (tailbone). The coccyx is a triangular-shaped bone in the lowermost region of the spine and is composed of three to five individual segments^1^. It serves as the attachment site for several pelvic structures and possesses an extensive nerve supply^2^. Coccydynia is five times more common in women than men, although its exact prevalence is unclear^3,4^. Factors associated with the development of coccydynia include obesity, prolonged sitting, and childbirth^1,4^. Degenerative joint disease is also thought to increase risk^1^. The most common presentation is following a traumatic event, typically a backwards fall^1^.

Most cases of coccydynia can be managed conservatively with rest, anti-inflammatory medications, seating aids, and warm baths^3^. Physiotherapy and manipulative therapy are also popular treatment options^5^. Steroid and anaesthetic injections may be useful in patients who do not respond to these methods, but there is controversy surrounding the optimal site for injection^1^. Surgical coccygectomy is performed in treatment-resistant cases, and whilst outcomes are generally good-to-excellent, the incision is made close to the anal canal and can make subsequent care challenging^6,7^.

Extracorporeal shockwave therapy (ESWT) is a physical agent modality that offers an alternative and non-invasive means of treatment for coccydynia^8^. Devices are placed against the skin and deliver impulses of energy to injured tissue at a given pressure (bar) and frequency (Hz)^9–11^. The exact mechanism of action of ESWT remains unknown; however, biological responses are proposed to be mediated by mechanotransduction and include tissue regeneration, wound healing, angiogenesis, and neovascularisation^12–15^. Altogether, this can result in significant pain alleviation^12^. There are two types of ESWT: focused (fESWT) and radial (rESWT). These differ marginally in terms of shockwave production and energy delivery^8^. In practice, rESWT devices are smaller, cheaper, and easier to use^8^. Moreover, rESWT has proven to be successful in the management of musculoskeletal disorders such as plantar fasciitis^16–18^, calcific shoulder tendinopathy^19,20^, trigger digit^21^, sesamoid osteonecrosis^22^, patellar tendinopathy^23^, medial tibial stress syndrome^24–26^, and lateral epicondylitis^27,28^.

Previous literature has highlighted the benefit of rESWT for coccydynia^29–32^. One study found rESWT to be more effective than steroid injections for pain relief over 6 months of observation^32^. A recent systematic review reported that shockwave therapy offered the second-greatest improvements in pain among all available treatment options^33^. However, research on the subject remains limited. Furthermore, most of the available studies adopt a ‘one size fits all’ approach by treating patients at a single dose over a predetermined number of sessions. The research objectives of this study were: (i) determine whether a pain-adapted protocol of rESWT is effective in treating coccydynia, (ii) assess for a relationship between treatment duration and symptom intensity, and (iii) monitor patients for possible adverse effects of treatment.

## Methods

This prospective study was reported in line with the PROCESS (Preferred Reporting Of CasE Series in Surgery) Guideline^34^, Supplemental Digital Content 1, http://links.lww.com/MS9/A206.

### Subjects

Patients presenting to a sports clinic located in Thessaloniki, Greece, between January 2017 and December 2020 were diagnosed with coccydynia by a consultant sports physician (N.M.) following focused clinical history, examination, and imaging. Physical examination included inspection of the overlying skin and palpation, which revealed localised focal tenderness in the region of the coccyx^3,35^. Anteroposterior and lateral radiographs of the lumbar spine and coccyx were obtained to assess the position of the coccyx and rule out coccygeal dislocations. Magnetic resonance imaging scans were also performed to exclude other pathologies of the lumbar spinal cord, coccyx, and associated soft tissue. Treatment options, including the use of rESWT, were discussed with patients if they reported a visual analogue scale (VAS) pain level ≥6. Patients aged ≥18 years were eligible for the study. Exclusion criteria were: local or systemic neurological disease, rheumatological disease, malignant disease, previous spinal surgery or spinal disease, coagulopathies, pregnancy, recent (≤6 weeks) trauma to the coccyx, and coccygeal dislocations.

### Equipment and procedure

Treatment was administered to the maximal point of tenderness exactly over and posterior to the coccyx by the same consultant sports physician, using the Storz Medical Masterpuls MP200 (Storz Medical; Tägerwilen, Switzerland) rESWT device^29,36^. All treatments were performed after verbal and written informed consent had been obtained. The probe was placed on the skin at 90° to the coccyx in the sagittal plane^29^. Ultrasound gel was applied to the skin before treatment to reduce energy loss from the device. Local anaesthesia is known to lower the efficacy of treatment and was therefore not used^37^. Treatment sessions took place once a week to allow for adequate periods of recovery and healing for at least three sessions. Any ongoing treatments for coccydynia were stopped, and patients were advised against the use of non-steroidal anti-inflammatory drugs.

The rESWT device was set to a minimum pressure and frequency of 1 bar and 5 Hz, respectively. Each session included the delivery of at least 2000 pulses. These parameters were set based on the experience of our authors with previous cases of coccydynia and mirror those utilised in similar rESWT research^16,19,21,22^. Pressure, frequency, and the number of pulses were adjusted between sessions in line with patients’ pain tolerance. Pain tolerance is the duration of time until an individual can no longer withstand an unpleasant stimulus and reflects the greatest level of discomfort they are willing to endure^38,39^. Tolerance is of particular value in coccydynia, given that pain is the most common presenting symptom and its management is the primary goal of treatment.

VAS pain levels were used to monitor patient response and clinical improvement throughout treatment, with patients ranking the severity of their pain at rest between ‘no pain’ (0) and ‘worst imaginable pain’ (10)^40,41^. Patients were asked their pain level before treatment at each session, although this did not form part of our formal data collection. Treatment continued until patients reported a VAS pain level ≤3, at which point they were discharged from the clinic^42^.

### Study outcomes

The main outcome measure of this study was pain management. Each patient provided a baseline VAS pain level based on their initial symptomology. Follow-up measurements were taken immediately after the cessation of treatment, at 3 months posttreatment, and at 12 months posttreatment. Symptom resolution and disease recurrence were monitored during this period. The mean device pressure and frequency, number of pulses, and number of rESWT sessions were also documented using electronic device records.

### Statistical analysis

Descriptive statistical analysis was carried out using Microsoft Excel (Microsoft Corporation, Redmond, Washington, USA), IBM SPSS Statistics Version 27 (IBM Corporation, Armonk, New York, USA), and Jamovi (Version 2.3, Sydney, Australia). The variables analysed did not meet the normality assumption, which was performed using the Shapiro–Wilk test (*P<*0.001). The repeated measures ANOVA (analysis of variance) procedure (Friedman) was used to test for significant reductions in VAS pain levels. Pairwise comparisons using Durbin–Conover measured differences in VAS at each stage of follow-up when compared with baseline scores. Spearman’s rank correlation coefficient was used to assess the correlation between variables. A predefined confidence level of 95% (*α*=0.05) was set for all statistical analyses.

## Results

Fourteen patients were included in the study. The cohort comprised five (36%) men and nine (64%) women, with a mean age of 33.6±7.9 (range: 20–45) years. The average duration of symptoms before rESWT was 9.4±8.5 (range: 3–36) months (Table 1).

**Table 1:** Patient characteristics.

Characteristic	Number of participants, *n* (%)
Sex	
Male	5 (36)
Female	9 (64)
Age (years)	
20–29	5 (36)
30–39	6 (43)
40–49	3 (21)
Duration of symptoms (months)	
3–6	7 (50)
7–12	6 (43)
>12	1 (7)

The mean number of rESWT sessions was 6.4±1.6 (range: 4–8). The median number of sessions was 6.5 (Q1: 5; Q3: 8). Five patients (36%) underwent eight sessions, two (14%) underwent seven sessions, two (14%) underwent six sessions, three (21%) underwent five sessions, and two (14%) underwent four sessions (Table 2). There was a strong correlation between the chronicity of symptoms and the number of rESWT sessions required (*r*=0.701, *P*=0.005).

**Table 2:** rESWT device parameters during treatment.

	Session 1 (*n*=14)	Session 2 (*n*=14)	Session 3 (*n*=14)	Session 4 (*n*=14)	Session 5 (*n*=12)	Session 6 (*n*=9)	Session 7 (*n*=7)	Session 8 (*n*=5)
Pressure (bar)								
Mean	1.1	1.1	1.2	1.3	1.3	1.3	1.3	1.3
Median	1.1	1.1	1.3	1.4	1.4	1.4	1.4	1.4
Frequency (Hz)								
Mean	5	5	5	5.2	5	5	5	5
Median	5	5	5	5	5	5	5	5
Number of pulses								
Mean	2000	2100	2100	2179	2175	2100	2000	2000
Median	2000	2100	2100	2250	2300	2000	2000	2000

The mean pressure and frequency of the rESWT device over the course of all treatments were 1.2±0.1 (range: 1–1.4) bar and 5.0±0.1 (range: 5–6) Hz, respectively. The median pressure and frequency was 1.2 (Q1: 1.1; Q3: 1.4) bar and 5.0 (Q1: 5.0; Q3: 5.0) Hz, respectively. The mean number of pulses delivered per session was 2082±74.8 (range: 2000–2300) pulses. The median number of pulses per session was 2000 (Q1: 2000; Q3: 2200) pulses (Table 2). No patient dropped out of the study and no adverse effects were described. A full breakdown of the rESWT protocols is attached in the supplementary material (Supplemental Digital Content 2, http://links.lww.com/MS9/A207).

**Figure 1:**
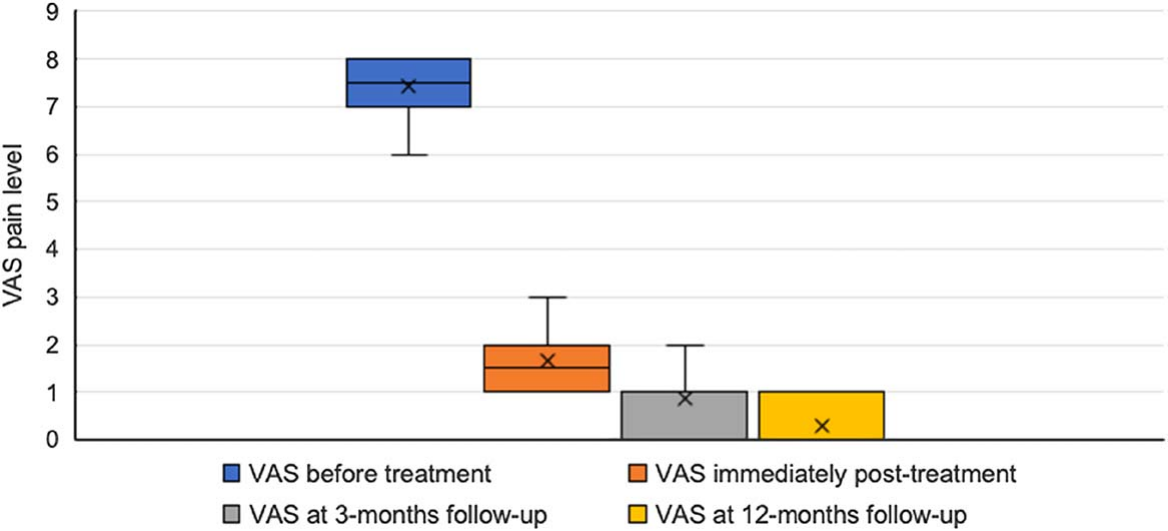
Box plot displaying median, quartile, minimum, and maximum VAS ratings at each stage of treatment. VAS, visual analogue scale.

The mean and median pretreatment VAS was 7.4±0.7 (range: 6–8) and 7.5 (Q1: 7, Q3: 8), respectively. The mean and median VAS immediately posttreatment was 1.6±0.7 (range: 1–3) and 1.5 (Q1: 1, Q3: 2), respectively. The mean and median VAS at 3-month follow-up were 0.9±0.7 (range: 0–2) and 1.0 (Q1: 0.25; Q3: 1), respectively. The mean and median VAS at 12-month follow-up was 0.3±0.5 (range: 0–1) and 0.0 (Q1: 0; Q3: 0.75), respectively (Fig. 1). A breakdown of individual VAS pain levels at each stage is available in the supplementary material (Supplemental Digital Content 2, http://links.lww.com/MS9/A207).

The nonparametric Friedman test of differences rendered a statistically significant chi-square (*χ* ^2^) value of 38.7 (*P*<0.001). The pairwise comparison revealed significant reductions in VAS pain levels between each stage of treatment (*P*<0.001) (Table 3). No recurrence of symptoms was reported during the follow-up period.

**Table 3:** Pairwise comparisons using Durbin–Conover.

			Statistic	*P*
VAS before treatment	–	VAS immediately posttreatment	9.0	<0.001
VAS before treatment	–	VAS at 3-month follow-up	15.1	<0.001
VAS before treatment	–	VAS at 12-month follow-up	20.4	<0.001
VAS immediately posttreatment	–	VAS at 3-month follow-up	6.1	<0.001
VAS immediately posttreatment	–	VAS at 12-month follow-up	11.3	<0.001
VAS at 3-month follow-up	–	VAS at 12-month follow-up	5.3	<0.001

VAS, visual analogue scale.

## Discussion

Our study recorded high success rates when treating coccydynia with pain-adapted protocols using rESWT. Mean VAS pain level reductions of 78%, 89%, and 96% were recorded immediately posttreatment, at 3-month, and 12-month follow-up, respectively, compared to baseline. Patients with longer-standing symptoms at presentation generally required more treatment sessions. There was no symptom recurrence reported during the follow-up.

The value of rESWT for coccydynia has been highlighted in a handful of studies^29–32^. However, across these studies, rESWT device pressure (2–4 bar), frequency (5–21 Hz), number of pulses (2000–3000 pulses), and number of sessions ranged considerably. To our knowledge, this study is the first to deliver a pain-adapted protocol of rESWT. We achieved this by (i) adjusting rESWT device parameters in line with patients’ pain tolerance and (ii) continuing treatment until a VAS pain level ≤3 was reported. We believe that continuing treatment until a low level of pain is achieved is relevant to the favourable outcome of this study.

Treating patients according to pain tolerance is common with the use of shockwave therapy. Numerous regimes in a 2013 systematic review described making dose alterations ‘as tolerated’ when treating soft tissue conditions^43^. To illustrate, if a patient found the same dose of treatment to be less painful at a later session compared to an earlier session, then device parameters may be increased. Alternatively, if a patient finds treatment uncomfortable at a particular dose, then device parameters may be lowered. A 2007 study also observed the greatest reductions in pain when patients with chronic heel pain were treated with ‘maximum tolerable’ energy densities, compared to a fixed protocol^44^.

The mean device pressure in this study increased by a maximum of 0.2 bar (1.1–1.3 bar). Whilst this may be an arguably insignificant change, it is worth noting the positive outcomes observed at lower overall device pressures^19^. Higher device pressures are said to increase pain; therefore, our approach may also make treatment more comfortable for patients^45^. The mean device frequency increased by a maximum of 0.2 Hz (5–5.2 Hz), and the mean number of pulses per session increased by a maximum of 8.95% (2000–2179 pulses). An increased number of larger cavitation bubbles – and resultant shockwaves – are generated at lower frequencies, which may explain the findings of one study, where the device frequency was set to 21 Hz, and increases in pain were reported at the 7-month follow-up^30,46^. In contrast, no symptom recurrence was present in our study. The number of pulses per session increased only between sessions numbers two and six, and with careful consideration of energy accumulation in those who had more than six sessions.

Patients were discharged from the clinic once their VAS pain level reached ≤3. This meant that they all underwent at least four, and no more than eight, treatment sessions. In contrast, previous studies administered treatment over a predetermined number of sessions^30,31^. Given the symptom control and lack of recurrence in our cohort, we may argue for the number of treatment sessions to be dictated by patients’ pain levels, as this appears to be the most important indicator of successful treatment. Pain levels after treatment and during follow-up were also lower in our cohort compared to others.

Yet, inadequate pain relief among patients was observed in another study that continued rESWT until VAS pain levels were ≤3^29^. Equally, we acknowledge that complete symptom resolution (VAS=0) was not achieved among our entire sample, and, in fact, no patient was completely pain-free immediately posttreatment (VAS 1-3). While the long-term effect of rESWT can be observed during follow-up, four patients remained symptomatic (VAS=1) at 12 months. This suggests that while treatment may have been successful, it did not always eradicate pain. A lengthier follow-up period may have highlighted further decreases or recurrences in VAS; however, 12 months can be considered adequate, given the nature and conditions of this study.

Our analyses found a strong positive correlation between symptom duration and the number of treatment sessions required (*r*=0.701). There were seven patients who underwent either seven or eight sessions, all of whom reported pain for more than 6 months. However, symptom duration did not appear to be reflected in pretreatment VAS. The remaining patients, whose symptomologies ranged from 3–6 months, required no more than six sessions. This suggests that patients with chronic symptoms (>6 months) at presentation are likely to require more rESWT sessions.

Despite its poorly understood mechanism of action, rESWT continues to serve as a safe and effective management option for coccydynia^47,48^. Based on our findings, the following treatment protocol may be proposed: 1.2 bar, 5 Hz, and 2000 pulses per session until patients report a VAS pain level ≤3. There appears to be value in adjusting treatment doses according to pain tolerance, both in optimising long-term pain management and minimising the risk of recurrence. This approach may also make rESWT more comfortable for patients. However, our research was not comparative; therefore, only tentative conclusions can be drawn. There is a need for randomised controlled trials (RCTs) comparing rESWT with other management options for coccydynia and comparing different rESWT protocols.

### Limitations

We acknowledge some limitations of this study. Firstly, the number of patients included was small because coccydynia is a condition of low incidence. Secondly, our analysis must be interpreted with some degree of caution. Although a single modality was delivered, we cannot be certain that there were no alternative explanations for the effects observed. Additionally, VAS pain levels during treatment were not formally recorded. VAS was also the only measure used to assess patients’ pain tolerance. Employing a second tool to confirm the decrement of intensity may have strengthened our conclusions regarding the efficacy of rESWT. Other outcomes, such as quality of life and disability, have featured in similar research and assessed using scales such as SF-36 and Oswestry Disability Index, respectively.

## Conclusion

We had identified a high success rate and no recurrence of disease at 12-month follow-up when coccydynia patients were treated with a pain-adapted protocol using rESWT. Our results align with the current evidence base recognising rESWT as a conservative management option for coccydynia and also suggest that adjusting the number of treatment sessions and doses during treatment in line with patients’ pain tolerance may optimise pain management and lower the risk of recurrence. This may also make treatment more comfortable for patients. More RCTs are needed to compare the effect of rESWT on other surgical and non-surgical treatment modalities.

## Ethical approval

Ethical approval was granted by the Thessaloniki Clinic Institutional Review Board (ART No. 2016-09).

## Consent

Written informed consent was obtained from the patient for publication and any accompanying images. A copy of the written consent is available for review by the Editor-in-Chief of this journal on request.

## Sources of funding

This paper received no specific grant from any funding agency in the public, commercial, or not-for-profit sectors. The authors affirm that this manuscript is an honest, accurate, and transparent account of the study being reported, that no important aspects of the study have been omitted and that any discrepancies from the study as planned (and, if relevant, registered) have been explained.

## Author contribution

N.M.: set up and carried out the study; K.S.L. drafted the manuscript. All authors contributed to the writing of the manuscript, revised the draft manuscript, and approved the final version.

## Conflicts of interest disclosure

The authors declare that they have no conflicts of interest.

## Research registration unique identifying number (UIN)

1. Name of the registry: ClinicalTrials.gov.
2. Unique identifying number or registration ID: NCT05753514.

1. Hyperlink to your specific registration (must be publicly accessible and will be checked): https://clinicaltrials.gov/ct2/show/NCT05753514.

## Guarantor

Kabir Singh Lota, E-mail: ha16657@qmul.ac.uk; Nikos Malliaropoulos, E-mail: contact@sportsmed.gr; Georgios Bikos, E-mail: bikosg77@yahoo.gr; Heinz Lohrer, E-mail: Heinz.Lohrer@esn-ortho.de.

## Data availability statement

The authors confirm that the data supporting the findings of this study are available within the article and its supplementary materials.

## Provenance and peer review

Not applicable.

## Supplementary Material

SUPPLEMENTARY MATERIAL

## References

[1] Garg B Ahuja K. Coccydynia – a comprehensive review on etiology, radiological features and management options. J Clin Orthop Trauma 2021;12:123–129. 33716437 10.1016/j.jcot.2020.09.025 PMC7920198

[2] Mostafa E Varacallo M. Anatomy, Back, Coccygeal Vertebrae. StatPearls. Published online 31 July 2021. 31751060

[3] Lirette LS Chaiban G Tolba R. Coccydynia: an overview of the anatomy, etiology, and treatment of coccyx pain. Ochsner J 2014;14:84. 24688338 PMC3963058

[4] Chen Y Huang-Lionnet JHY Cohen SP. Radiofrequency ablation in coccydynia: a case series and comprehensive, evidence-based review. Pain Med 2017;18:1111–1130. 28034983 10.1093/pm/pnw268

[5] Elkhashab Y Ng A. A review of current treatment options for coccygodynia. Curr Pain Headache Rep 2018;22:28. 29556817 10.1007/s11916-018-0683-7

[6] Hanley EN Ode G Seymour R. Coccygectomy for patients with chronic coccydynia: a prospective, observational study of 98 patients. Bone Jt J 2016;98B:526–533. 10.1302/0301-620X.98B4.36641 27037436

[7] Kulkarni AG Tapashetti S Tambwekar VS. Outcomes of coccygectomy using the “Z” plasty technique of wound closure. Global Spine J 2019;9:802–806. 31819844 10.1177/2192568219831963 PMC6882096

[8] Lohrer H Nauck T Korakakis V. Historical ESWT paradigms are overcome: a narrative review. BioMed Res Int 2016;2016:e3850461. 10.1155/2016/3850461 PMC4967434 27493955

[9] Wang CJ. Extracorporeal shockwave therapy in musculoskeletal disorders. J Orthop Surg 2012;7:11. 10.1186/1749-799X-7-11 PMC3342893 22433113

[10] Verstraelen FU In den Kleef NJHM Jansen L. High-energy versus low-energy extracorporeal shock wave therapy for calcifying tendinitis of the shoulder: which is superior? A meta-analysis. Clin Orthop Relat Res 2014;472:2816–2825. 24872197 10.1007/s11999-014-3680-0 PMC4117900

[11] Melzack R Wall PD. Pain mechanisms: a new theory. Science 1965;150:971–979. 5320816 10.1126/science.150.3699.971

[12] Simplicio CL Purita J Murrell W. Extracorporeal shock wave therapy mechanisms in musculoskeletal regenerative medicine. J Clin Orthop Trauma 2020;11(Suppl 3):S309–S318. 32523286 10.1016/j.jcot.2020.02.004 PMC7275282

[13] Vetrano M d’Alessandro F Torrisi MR. Extracorporeal shock wave therapy promotes cell proliferation and collagen synthesis of primary cultured human tenocytes. Knee Surg Sports Traumatol Arthrosc 2011;19:2159–2168. 21617986 10.1007/s00167-011-1534-9

[14] Mittermayr R Hartinger J Antonic V. Extracorporeal shock wave therapy (ESWT) minimizes ischemic tissue necrosis irrespective of application time and promotes tissue revascularization by stimulating angiogenesis. Ann Surg 2011;253:1024–1032. 21372687 10.1097/SLA.0b013e3182121d6e

[15] Lohrer H Nauck T. Shock Waves in Sports Medicine, 1st ed. Level 10; 2018.

[16] Malliaropoulos N Crate G Meke M. Success and recurrence rate after radial extracorporeal shock wave therapy for plantar fasciopathy: a retrospective study. BioMed Res Int 2016;2016:9415827. 27478843 10.1155/2016/9415827 PMC4949339

[17] Sun J Gao F Wang Y. Extracorporeal shock wave therapy is effective in treating chronic plantar fasciitis. Medicine (Baltimore) 2017;96:e6621. 28403111 10.1097/MD.0000000000006621 PMC5403108

[18] Gerdesmeyer L Frey C Vester J. Radial extracorporeal shock wave therapy is safe and effective in the treatment of chronic recalcitrant plantar fasciitis: results of a confirmatory randomized placebo-controlled multicenter study. Am J Sports Med 2008;36:2100–2109. 18832341 10.1177/0363546508324176

[19] Malliaropoulos N. Individualised radial extracorporeal shock wave therapy (rESWT) for symptomatic calcific shoulder tendinopathy: a retrospective clinical study. BMC Musculoskelet Disord 2017;18:513. 29207984 10.1186/s12891-017-1873-x PMC5718020

[20] Cosentino R Stefano RD Selvi E. Extracorporeal shock wave therapy for chronic calcific tendinitis of the shoulder: single blind study. Ann Rheum Dis 2003;62:248–250. 12594112 10.1136/ard.62.3.248 PMC1754476

[21] Malliaropoulos N Jury R Pyne D. Radial extracorporeal shockwave therapy for the treatment of finger tenosynovitis (trigger digit. Open Access J Sports Med 2016;7:143–151. 27843364 10.2147/OAJSM.S108126 PMC5098764

[22] Thompson D Malliaropoulos N Padhiar N. Sesamoid osteonecrosis treated with radial extracorporeal shock wave therapy. BMJ Case Rep 2017;2017:bcr-2017-219191. 10.1136/bcr-2017-219191 PMC5753706 28536215

[23] van Leeuwen MT Zwerver J van den Akker-Scheek I. Extracorporeal shockwave therapy for patellar tendinopathy: a review of the literature. Br J Sports Med 2009;43:163–168. 18718975 10.1136/bjsm.2008.050740

[24] Rompe JD Cacchio A Furia JP. Low-energy extracorporeal shock wave therapy as a treatment for medial tibial stress syndrome. Am J Sports Med 2010;38:125–132. 19776340 10.1177/0363546509343804

[25] Korakakis V Whiteley R Tzavara A. The effectiveness of extracorporeal shockwave therapy in common lower limb conditions: a systematic review including quantification of patient-rated pain reduction. Br J Sports Med 2018;52:387–407. 28954794 10.1136/bjsports-2016-097347

[26] Moen MH Rayer S Schipper M. Shockwave treatment for medial tibial stress syndrome in athletes; a prospective controlled study. Br J Sports Med 2012;46:253–257. 21393260 10.1136/bjsm.2010.081992

[27] Król P Franek A Durmała J. Focused and radial shock wave therapy in the treatment of tennis elbow: a pilot randomised controlled study. J Hum Kinet 2015;47:127–135. 26557197 10.1515/hukin-2015-0068 PMC4633248

[28] Zheng C Zeng D Chen J. Effectiveness of extracorporeal shock wave therapy in patients with tennis elbow: a meta-analysis of randomized controlled trials. Medicine (Baltimore) 2020;99:e21189. 32791694 10.1097/MD.0000000000021189 PMC7387053

[29] Gönen Aydin C Örsçelik A Gök MC. The efficacy of extracorporeal shock wave therapy for chronic coccydynia. Med Princ Pract 2020;29:444–450. 31918431 10.1159/000505835 PMC7511685

[30] Haghighat S Asl MM. Effects of extracorporeal shock wave therapy on pain in patients with chronic refractory coccydynia: a quasi-experimental study. Anesthesiol Pain Med 2016;6:37428. 10.5812/aapm.37428 PMC5098426 27843777

[31] Lin SF Chen YJ Tu HP. The effects of extracorporeal shock wave therapy in patients with coccydynia: a randomized controlled trial. PLoS One 2015;10:e0142475. 26556601 10.1371/journal.pone.0142475 PMC4640534

[32] Ahadi T Hosseinverdi S Raissi G. Comparison of extracorporeal shockwave therapy and blind steroid injection In patients with coccydynia: a randomized clinical trial. Am J Phys Med Rehabil 2022;101:417–422. 34091468 10.1097/PHM.0000000000001802

[33] Andersen GØ Milosevic S Jensen MM. Coccydynia – the efficacy of available treatment options: a systematic review. Glob Spine J 2022;12:1611–1623. 10.1177/21925682211065389 PMC9393997 34927468

[34] Agha RA Sohrabi C Mathew G. The PROCESS 2020 guideline: updating consensus Preferred Reporting Of CasE Series in Surgery (PROCESS) guidelines. Int J Surg Lond Engl 2020;84:231–235. 10.1016/j.ijsu.2020.11.005 33189880

[35] Mabrouk A Alloush A Foye P. Coccyx Pain. StatPearls. StatPearls Publishing; 2021. [Accessed 6 October 2021]. http://www.ncbi.nlm.nih.gov/books/NBK563139/33085286

[36] Marwan Y Husain W Alhajii W. Extracorporeal shock wave therapy relieved pain in patients with coccydynia: a report of two cases. Spine J 2014;14:e1–e4. 10.1016/j.spinee.2013.07.438 24094989

[37] Rompe J Meurer A Nafe B. Repetitive low-energy shock wave application without local anesthesia is more efficient than repetitive low-energy shock wave application with local anesthesia in the treatment of chronic plantar fasciitis. J Orthop Res 2005;23:931–941. 16023010 10.1016/j.orthres.2004.09.003

[38] Martin RA Martin RA. Chapter 10 – Humor and Physical Health. The Psychology of Humor. Academic Press; 2007:309–333.

[39] Clark WC Chokhavatia SS Kashani A Argoff CE McCleane G. Chapter 6 – Pain Measurement. Pain Management Secrets, 3rd ed. Mosby; 2009:42–52.

[40] Hawker GA Mian S Kendzerska T. Measures of adult pain: Visual Analog Scale for Pain (VAS Pain), Numeric Rating Scale for Pain (NRS Pain), McGill Pain Questionnaire (MPQ), Short-Form McGill Pain Questionnaire (SF-MPQ), Chronic Pain Grade Scale (CPGS), Short Form-36 Bodily Pain Scale (SF-36 BPS), and Measure of Intermittent and Constant Osteoarthritis Pain (ICOAP). Arthritis Care Res (Hoboken) 2011;63(S11):S240–S252. 22588748 10.1002/acr.20543

[41] Delgado DA Lambert BS Boutris N. Validation of digital visual analog scale pain scoring with a traditional paper-based visual analog scale in adults. J Am Acad Orthop Surg Glob Res Rev 2018;2:e088. 30211382 10.5435/JAAOSGlobal-D-17-00088 PMC6132313

[42] Kelly AM. The minimum clinically significant difference in visual analogue scale pain score does not differ with severity of pain. Emerg Med J 2001;18:205–207. 11354213 10.1136/emj.18.3.205 PMC1725574

[43] Speed C. A systematic review of shockwave therapies in soft tissue conditions: focusing on the evidence. Br J Sports Med 2014;48:1538–1542. 23918444 10.1136/bjsports-2012-091961

[44] Chow IHW Cheing GL. Comparison of different energy densities of extracorporeal shock wave therapy (ESWT) for the management of chronic heel pain. Clin Rehabil 2007;21:131–141. 17264107 10.1177/0269215506069244

[45] Lee SJ Kang JH Kim JY. Dose-related effect of extracorporeal shock wave therapy for plantar fasciitis. Ann Rehabil Med 2013;37:379–388. 23869336 10.5535/arm.2013.37.3.379 PMC3713295

[46] Császár NBM Angstman NB Milz S. Radial shock wave devices generate cavitation. PLoS One 2015;10:e0140541. 26509573 10.1371/journal.pone.0140541 PMC4625004

[47] Schmitz C. Focused and radial extracorporeal shock wave therapy: more similarities than differences. Physiotherapy 2015;101:e1346–e1347.

[48] Romeo P Lavanga V Pagani D. Extracorporeal shock wave therapy in musculoskeletal disorders: a review. Med Princ Pract 2014;23:7–13. 24217134 10.1159/000355472 PMC5586835

